# Two-view topogram-based anatomy-guided CT reconstruction for prospective risk minimization

**DOI:** 10.1038/s41598-024-59731-y

**Published:** 2024-04-23

**Authors:** Chang Liu, Laura Klein, Yixing Huang, Edith Baader, Michael Lell, Marc Kachelrieß, Andreas Maier

**Affiliations:** 1 Pattern Recognition Lab, Friedrich-Alexander-Universität Erlangen-Nürnberg (FAU), Erlangen, Germany; 2 Division of X-Ray Imaging and Computed Tomography, German Cancer Research Center (DKFZ), Heidelberg, Germany; 3 Department of Physics and Astronomy, Ruprecht-Karls-University Heidelberg, Heidelberg, Germany; 4 Medical Faculty, Ruprecht-Karls-University Heidelberg, Heidelberg, Germany; 5 Department of Radiology and Nuclear Medicine, Klinikum Nürnberg, Paracelsus Medical University, Nuremberg, Germany; 6 Department of Radiation Oncology, Universitätsklinikum Erlangen, Friedrich-Alexander-Universität Erlangen-Nürnberg (FAU), Erlangen, Germany

**Keywords:** CT, Two-view reconstruction, Computed tomography, Radiography

## Abstract

To facilitate a prospective estimation of the effective dose of an CT scan prior to the actual scanning in order to use sophisticated patient risk minimizing methods, a prospective spatial dose estimation and the known anatomical structures are required. To this end, a CT reconstruction method is required to reconstruct CT volumes from as few projections as possible, i.e. by using the topograms, with anatomical structures as correct as possible. In this work, an optimized CT reconstruction model based on a generative adversarial network (GAN) is proposed. The GAN is trained to reconstruct 3D volumes from an anterior-posterior and a lateral CT projection. To enhance anatomical structures, a pre-trained organ segmentation network and the 3D perceptual loss are applied during the training phase, so that the model can then generate both organ-enhanced CT volume and organ segmentation masks. The proposed method can reconstruct CT volumes with PSNR of 26.49, RMSE of 196.17, and SSIM of 0.64, compared to 26.21, 201.55 and 0.63 using the baseline method. In terms of the anatomical structure, the proposed method effectively enhances the organ shapes and boundaries and allows for a straight-forward identification of the relevant anatomical structures. We note that conventional reconstruction metrics fail to indicate the enhancement of anatomical structures. In addition to such metrics, the evaluation is expanded with assessing the organ segmentation performance. The average organ dice of the proposed method is 0.71 compared with 0.63 for the baseline model, indicating the enhancement of anatomical structures.

## Introduction

Computed tomography (CT) imaging provides non-invasive insights into the human body with a high image quality and only short acquisition time compared to other modalities. Therefore, CT imaging has become an integral part of clinical routine and research. However, in order to reconstruct CT volumes with a diagnostic image quality, a sufficient number of measured projections must be acquired which inevitably exposes the patient to ionizing radiation, i.e., X-rays. Therefore, dose reduction is an important research topic in CT imaging. There are different methods to achieve dose reduction, both hardware- and software-based. These methods include but are not limited to the usage of pre-filters, iterative reconstruction algorithms, and dose-shielding methods. One other method that is routinely used is to adjust the tube current of the X-ray source depending on the angular position $\alpha$ of the X-ray source and the z-position, so called tube current modulation (TCM)^1,2^. More precisely, TCM methods aim at minimizing the mAs-product by adapting the tube current as a function of attenuation for a given view. The attenuation can for example be estimated based on the topogram acquired prior to the CT scan.

However, the mAs-product is only a surrogate parameter for actual patient dose, since some organs are more sensitive to the radiation than others. It would be of advantage to also account for these sensitivities in the tube current optimization. Thereby, the effective dose $D_{\text {eff}}$ is defined as the sum of the dose absorbed by the organ-at-risks (OAR) during the exposure, weighted with the organ-specific tissue weighting factor. The tissue weighting factors corresponding to the radiation sensitivity of the individual organs and structures are provided by the international commission on radiological protection (ICRP)^3,4^. The factors also reflect the risk of radiation induced cancer. Recently, a risk-minimizing tube current modulation (riskTCM) has been proposed that requires a dose distribution for every view and the organ segmentation as input parameters^5^ and is then able to minimize $D_{\text {eff}}$ for the actual CT scan. In particular, this method assumes an initial coarse CT reconstruction and the voxel-wise segmentation of all relevant organs. Given the known sensitivities with respect to ionizing radiation of these organs, the effective dose is estimated on a per-view basis. Usually, dose estimation is performed using Monte Carlo methods. Such methods, however, are very time consuming and would prohibit an application of riskTCM in clinical practice. Hence, spatial dose distribution is estimated in quasi-real-time from a given CT volume using a deep neural network proposed by Maier et al.^6^. Organ doses are then obtained using the known organ segmentation. With the effective dose for each potential view in the desired scan range, a tube current curve is then computed that allows maintaining diagnostic image quality while minimizing the patient risk.

**Figure 1:**
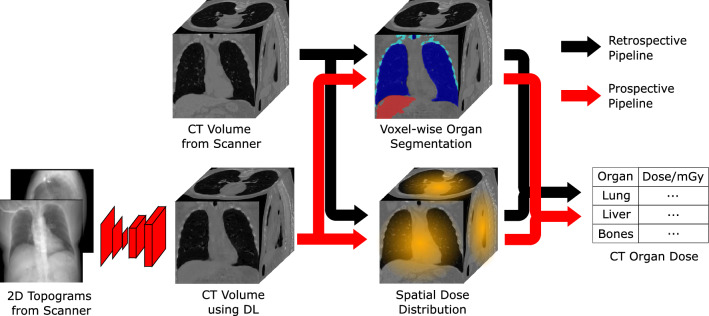
Illustration of prospective and retrospective CT organ dose estimation pipelines. Many existing methods to estimate CT organ doses are designed as retrospective pipelines, which can only be applied after the scanning. For applications like risk minimizing TCM methods for CT, a prospective pipeline for organ dose estimation is required. We propose to leverage a neural network to reconstruct the CT volume from the few X-ray projections to fasciliate such a prospective pipeline.

To achieve this, a method that estimates a coarse CT reconstruction before the scanning is needed. As shown in Fig. 1, starting from only few projections provides a reasonable pipeline to facilitate the CT risk optimization rather than the retrospective CT dose estimation. In order to avoid additional X-ray projections, we refactor the research problem to the reconstruction of a coarse CT volume from only two orthogonal topograms, referred to as X-ray projections in the following manuscript. One or two topograms are acquired before every CT scan and, therefore, do not add extra radiation exposure to the patient.

With the emerge of deep learning (DL)-based medical image processing methods, some generative adversarial network (GAN) methods have been established related with CT reconstruction from only few views^7^. Ying et al. proposed X2CT-GAN^8^ that performs a domain transfer task from X-ray projections to CT volumes, where a network for effective 2D-to-3D image generation is proposed. The authors also address the superiority of using two X-ray projections, i.e. from anterior-posterior (a.p.) and lateral (lat.) direction, compared to only a single view. On top of the X2CT-GAN, Ling et al. proposed a conditional variational autoencoder (cVAE)-based GAN^9^ to enhance the regularization of the generator. Ratul et al. improved the generator with additional input of the organ segmentation of the X-ray projections from a.p. direction^10^. Montoya et al. proposed ScoutCT-Net that first backprojects the topograms into an initial CT volume, and refine the initial volume using another network^11^. Similarly, most proposed methods aim to improve the CT reconstructions by voxel-wise metrics, while the anatomical information, such as the shape and location of organs and structures, are usually ignored.

In this work, we propose an anatomy-guided GAN for CT reconstruction from only two X-ray projections which can facilitate the implementation of risk-specific TCM methods. More specifically, a 3D perceptual loss $L_p$ and a 3D segmentation loss $L_s$ are implemented into the overall loss function for training the GAN, leading to a loss function that also optimizes for better anatomical information:

1 $$\begin{aligned} L_G' = L_G + \lambda _p L_p + \lambda _s L_s, \end{aligned}$$

where $L_G$ is the original generator loss, focusing on voxel-wise similarity, and $\lambda _p$ and $\lambda _s$ are constants that control the enhancement. We demonstrate that the combined use of $L_p$ and $L_s$ can lead to the enhancement of anatomical structures in the reconstructed volumes. The implementation of $L_p$ and $L_s$ will be in detail explained in following sections. Our proposed method enhances the organ shapes and boundaries during the training phase and thus will not increase the computational complexity during inference time.

## Results

**Figure 2:**
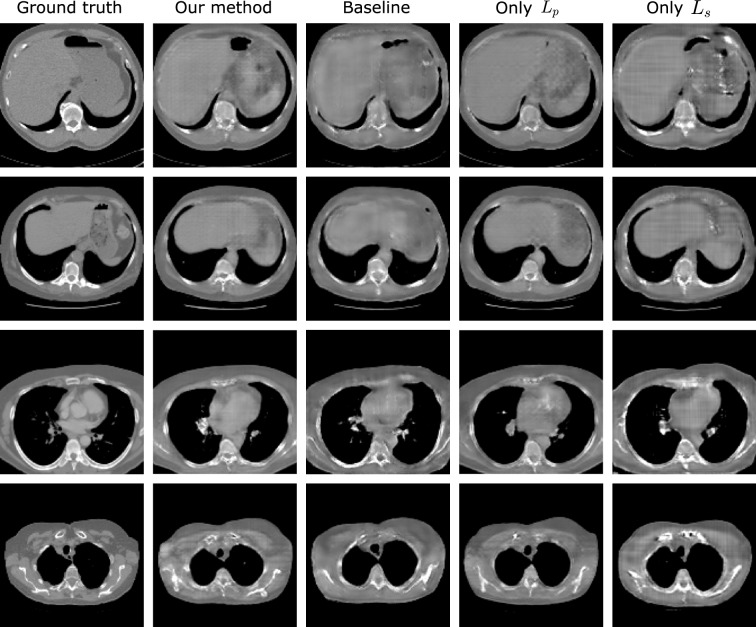
Exemplary slices of the reconstructed CT volumes using the proposed and the baseline method, in comparison with the ground truth. $L_p$ indicates the proposed 3D perceptual loss and $L_s$ the 3D segmentation loss. Slices of each row are drawn from four different volumes in the test set.

**Figure 3:**
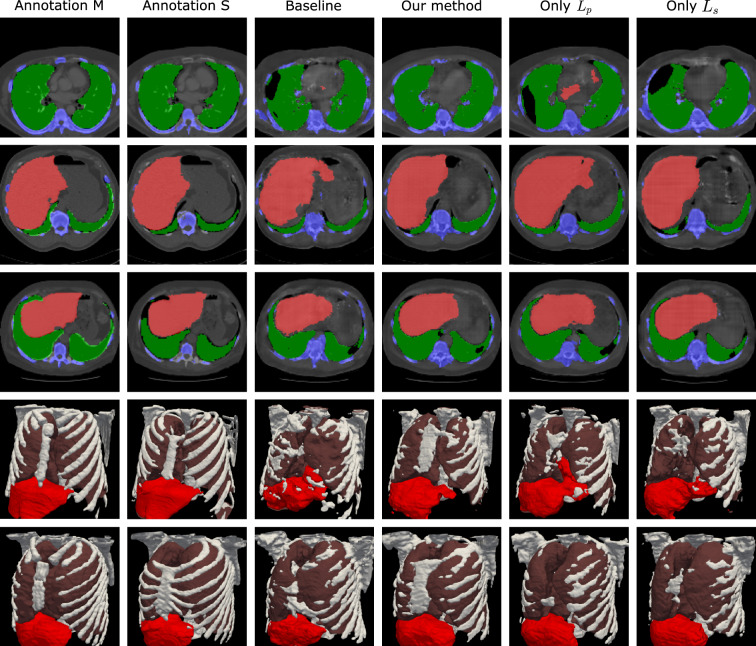
Comparison of the organ segmentation masks generated in different experiments. In the top three rows, the slices of the organ segmentation mask and the CT volumes are shown. In the bottom rows the organ segmentation of the volumes in the first two rows is shown as mesh visualization.

Some exemplary slices of the reconstructed CT volumes are shown in Fig. 2 and the results of organ segmentation are shown in Fig. 3. The X2CT-GAN by Ying et al.^8^ is implemented as the baseline model and our proposed methods are evaluated in comparison in terms of the anatomical structures. After analysis of the reconstruction performance, we choose $\lambda _s=2.0$ and $\lambda _p=0.5$ to present the reconstruction performance of our proposed method. We demonstrate that our proposed method can improve both the overall image quality and the anatomical structures, in comparison with the baseline method. More specifically, $L_p$ leads to the improved image quality while $L_s$ can improve the anatomical plausibility of organs and structures. The influence of the $L_p$ and $L_s$ are further investigated in the ablation experiments, where either $L_p$ or $L_s$ is applied for enhancement with varying $\lambda$s.

**Table 1:** Reconstruction and organ segmentation results of the experiments.

	Baseline		Proposed method		Only $L_p$		Only $L_s$	
	Avg.	Std.	Avg.	Std.	Avg.	Std.	Avg.	Std.
PSNR/dB	26.21	1.02	26.49	1.29	26.53	1.03	25.52	1.19
SSIM	0.62	0.06	0.64	0.05	0.64	0.05	0.60	0.06
RMSE/HU	201.55	24.22	196.17	31.63	195.63	33.29	219.28	33.30
$\text{DSC}_\text{M}$	0.63	0.02	0.71	0.03	0.69	0.02	0.70	0.01
$\text{DSC}_\text{S}$	0.71	0.03	0.76	0.03	0.74	0.03	0.76	0.02

$\text{DSC}_{\text{M}}$ indicates the DSC with manual annotation as ground truth and $\text{DSC}_{S}$ indicates the DSC with the segmentation using pre-trained segmentation network as ground truth. The segmentation metrics are aggregated from 20 CT volumes.

**Table 2:** Organ-wise segmentation results of the proposed method in comparison with the baseline method.

	Baseline				Proposed Method			
	Liver	Lung	Bones	Avg.	Liver	Lung	Bones	Avg.
$\text{DSC}_\text{M}$	0.74	0.81	0.35	0.63	0.82	0.86	0.45	0.71
$\text{DSC}_\text{S}$	0.75	0.84	0.57	0.71	0.81	0.86	0.63	0.76

### Reconstruction performance

**Figure 4:**
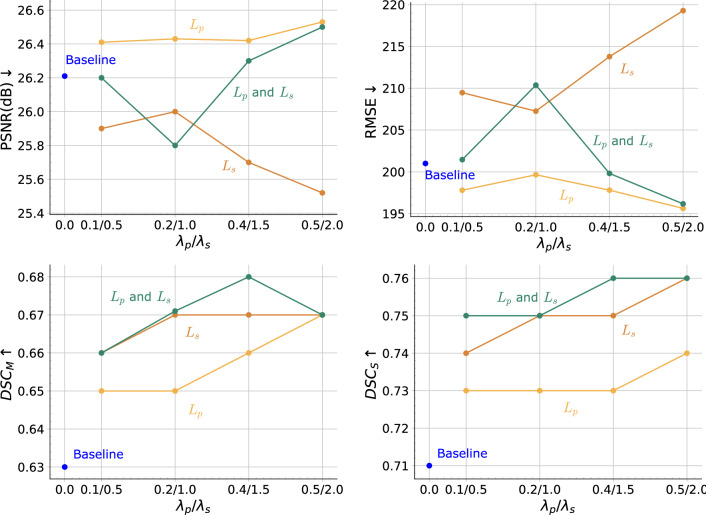
Reconstruction and organ segmentation performance of the proposed method with varying $\lambda$, in comparison with applying $L_p$ and $L_s$ independently.

From the exemplary slices in Fig. 2. The reconstructed CT volumes from the baseline method are ’visual real’ but only in terms of the body shape and regions such as the boundary of the lungs. However, the abdominal organs, for example the liver, are not distinctive from the remainder regions. Some structural details, like the shape of vertebra, are also lost. The proposed method leads to the enhancement of such anatomical structural details and the organ contrast, while keeping the overall image quality. More specifically, the application of $L_s$ decreases the image quality. This is expected as $L_s$ only enhances the organ segmentation rather than the voxel-wise image quality. Regarding the anatomy, $L_s$ contributes to organ-specific enhancement. As $L_s$ in our experiments only targets for lung, liver and bones segmentation, the contrast of such organs in the reconstructed CT volumes are enhanced while there is no enhancement for other organs and structures. With $L_p$ the anatomical structures in the CT volumes are enhanced, such as the shape of vertebrae, and the contrast between adjacent anatomical structures, such as the boundary of the fat tissues. However, such anatomical improvements are barely indicated by the reconstruction metrics. Peak-signal-to-noise ratio (PSNR), structural similarity index (SSIM) and root mean squared error (RMSE) in the unit of Hounsfield unit (HU) are selected to evaluate the reconstruction performance. Table 1 shows the results of our proposed method in comparison with the baseline method. Our proposed method leads to the improvement in the PSNR by 1.0% and the SSIM by 3.2%, and RMSE by 2.7%. With only $L_s$ , the PSNR is deteriorated by 2.6%, SSIM by 3.2% and RMSE by 8.7%. The best improvement in metrics is obtained for only $L_p$ , with an improvement for PSNR by 1.2%, for SSIM by 3.2% and for RMSE by 2.9%. From the reconstruction metrics, only $L_p$ can contribute to the improved image quality. The results from the ablation study are shown in Fig. 4. Here, higher $\lambda _p$ can lead to higher PSNR and SSIM, indicating higher overall image quality. In contrast, higher $\lambda _s$ will not improve the overall image quality. The proposed method also results in higher PSNR and RMSE when $\lambda _s$ and $\lambda _p$ increase, similar to the results with only $L_p$ .

#### Organ segmentation in reconstruction

In addition to the reconstruction metrics, we also evaluated the organ segmentation of the reconstructed volumes for assessment of the human anatomy. In our experiments, the segmentation of liver, lung and bones are evaluated, as defined by $L_s$ . Since the reconstruction dataset contains no paired organ segmentation annotation, 20 CT volumes in the test set are manually annotated with such organs, namely annotation *M*. In addition to the manual ground truth, the organ segmentation masks by the pre-trained segmentation network of $L_s$ are also used to benchmark the segmentation performance, named as annotation *S*. The dice similarity coefficient (DSC) of each organ is then computed.

Evaluated using the annotation *M*, the proposed method leads to the increase by 12.6% in average DSC compared to the baseline method, 9.5% when only $L_p$ is applied and 11.1% when only $L_s$ is applied. When the annotation *S* is used as ground truth, the proposed method leads to the increase by 7.0% in average DSC compared with the baseline method, an increase of 4.2 % when only $L_p$ is applied and 7.0 % when only $L_s$ is applied. In terms of each single organ, as shown in Table 2, the proposed method improves the $DSC_M$ by 15.1% for bones, 10.9% by liver and 6.1% by lungs. Also the $DSC_S$ is increased by 8.0% for liver and 10.5% for bones.

Some exemplary organ segmentation masks are shown in Fig. 3. The organ segmentation using the proposed method shows higher anatomical plausibility in terms of the organ and skeleton shape, as shown by the mesh visualization in Fig. 3. In comparison, the baseline method and the model with only $L_p$ contains more outliers and the segmentation of the skeleton is less accurate. As also shown in Fig. 4, higher $\lambda _s$ in general leads to higher average $\text{DSC}_{M}$ and $\text{DSC}_{S}$.

## Discussion

Based on the results, the proposed $L_p$ and $L_s$ contribute to the enhancement of both the anatomical structures and the overall image quality. Such enhancements enable the GANs to reconstruct CT volumes that not only appears correct but also ensures the reliability of the anatomical structures in the reconstructed volumes. Consequently, a more robust reconstruction method for a prospective pipeline for a risk-minimizing TCM for CT is established. However, the accurate inference of the radiation risk involves more organs than liver, lungs and bones in our research, and the whole human body should be included. Therefore, in our future research we aim to include more relevant OARs and whole body CTs.

It can be observed in Table 1 and Table 2 that the $DSC_S$ is always higher than the $DSC_M$. The potential explanation is that the predicted organ segmentation and the mask *M* are both generated by the same pre-trained segmentation network. It is intuitive that $L_s$ can lead to the increased $DSC_S$ as the GAN training is also optimized to minimize $L_s$. Subsequently, it is shown that the increase in $DSC_S$ is accompanied by an increase in $DSC_M$, indicating that the anatomical structures in the reconstructed volumes are actually enhanced.

**Figure 5:**
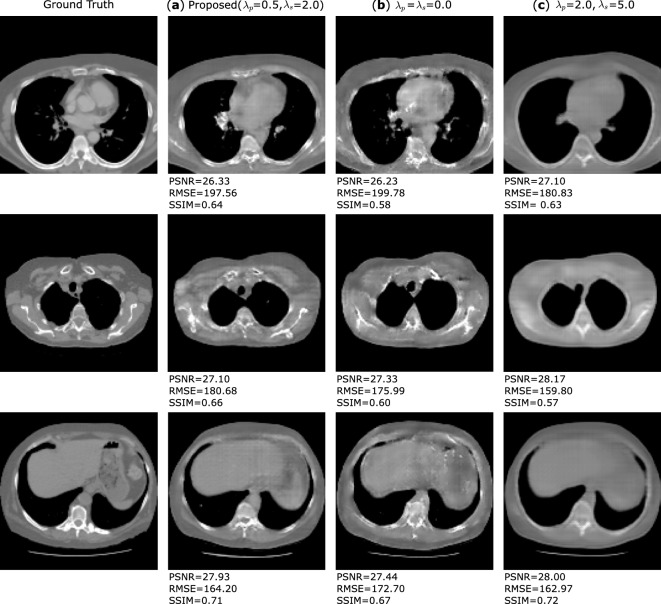
Example slices for which GANs fails to reconstruct the anatomical structures in the CT volumes. Column (a) shows the results for our proposed method with enhanced anatomical structures. In column (b) and (c) we present some reconstructed CT volumes each from the baseline model ($\lambda _S=\lambda _P=0.0$) and a over-enhanced model ($\lambda _S=5.0$, $\lambda _P=2.0$), where the GAN reconstructs the CT volumes with deteriorated anatomical structure but high reconstruction metrics.

Throughout our investigation, we have noted that the reconstructed volumes with enhanced anatomical structures can lead to inferior reconstruction metrics, i.e. PSNR, SSIM and RMSE. PSNR and RMSE are commonly used for the evaluation of reconstruction algorithms, and SSIM is originally designed for the assessment of digital image quality. Different from typical CT reconstruction methods, GAN-based methods depend on training a generator network to reconstruct the volumes from bi-planar projections, so such reconstruction is an ill-posed problem. During the training of the GANs, the network tends to reconstruct the CT volumes with bare or even no anatomical information, while maintaining high reconstruction metrics such as PSNR and SSIM. Some exemplary slices are shown in Fig. 5. Therefore, in our research we also evaluate the organ segmentation of the CT volumes, based on the assumption that a network that is trained for organ segmentation can effectively evaluate anatomical structures.

## Methods

**Figure 6:**
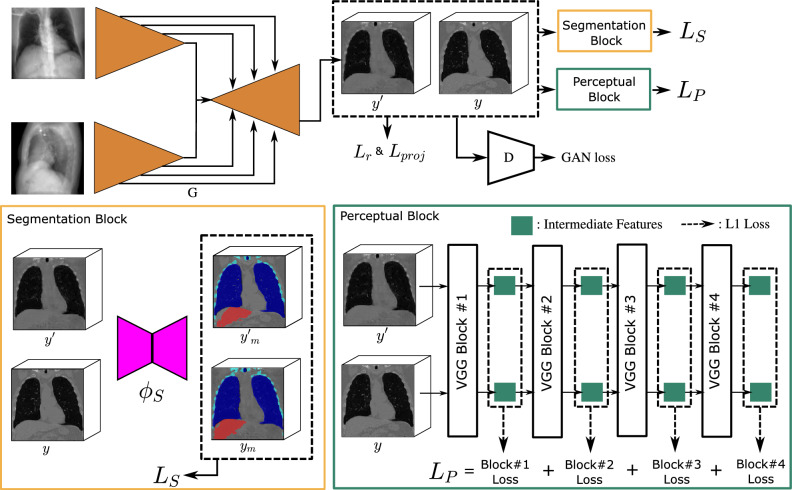
The proposed CT reconstruction pipeline with enhancement of anatomical structures. The CT Gen is the CT generator network that reconstructs the 3D CT volume with two 2D X-ray projections as input. The SegNet is a pre-trained segmentation network that segments the target anatomical structures and is frozen during the GAN training. The 3D perceptual loss aggregates the 2D perceptual losses from slices along vertical directions.

The pipeline of the proposed model is shown in Fig. 6. A GAN is trained to reconstruct a CT volume from two X-ray projections. On top of the typical generator and discriminator network of GAN^7^, two pre-trained networks are included into the training procedure, i.e. a pre-trained segmentation network, namely $\phi _s$, for the enhancement of specific anatomical structures and a pre-trained VGG network for the enhancement of the image quality^12^. VGG network is firstly proposed by the visual geometry group (VGG) from the university of Oxford and is a well-known network for image feature extraction in computer vision researches.

### CT reconstruction GAN

**Figure 7:**
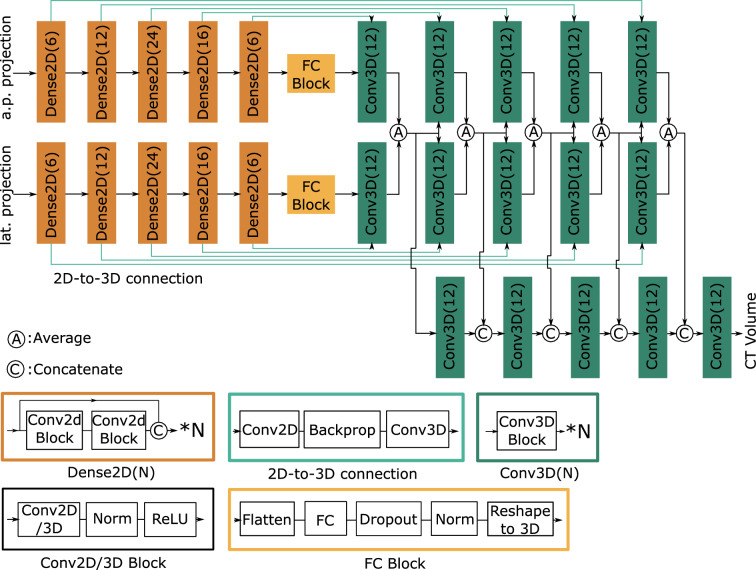
Network architecture of the CT generator network with 2D X-ray projections as input and 3D CT volumes as output.

The training of our proposed model follows the adversarial strategy of GAN. The minmax objective of GAN training in our situation is^7^

2 $$\begin{aligned} \underset{G}{\text{min}}\,\underset{D}{\text{max}}\,L_{\text{GAN}} = \mathbb {E}_y[\log D(y)] + \mathbb {E}_x[\log (1-D(G(x))], \end{aligned}$$

where *x* indicates the input X-ray projections and *y* the corresponding CT volume, $G(x;\theta _g)$ and $D(y;\theta _d)$ are the generator and discriminator network. $y'=G(x)$ is the reconstructed volume from only two X-ray projections. $\mathbb {E}$ indicates the function for mean value. More specifically, the GAN loss is modified according to least squared GAN as two loss functions^13^

3 $$\begin{aligned}&L_{\text{dis}}(x, y) = \frac{1}{2}(\mathbb {E}_{y}\left\| D(y)-1\right\| _2^2+\mathbb {E}_{x}\left\| D(G(x))-0\right\| _2^2), \end{aligned}$$

4 $$\begin{aligned}&L_{\text{gen}}(x) = \mathbb {E}_{x}\left\| D(G(x))-1)\right\| _2^2. \end{aligned}$$

In our model, the discriminator network is implemented as in the work of Phillip et al.^14^. We leverage the generator network of the X2CT-GAN by Ying et al. in our model^8^. The generator network encodes the input 2D X-ray projections using two independent pathways based on U-Net^15^. At each upsampling level of the U-Net pathways, the 3D features corresponding to the two topograms are fused by addition, and another 3D decoder aggregates the fused 3D features to output the reconstructed CT volume in 3D. The network architecture of the generator network is shown in Fig. 7.

**Figure 8:**
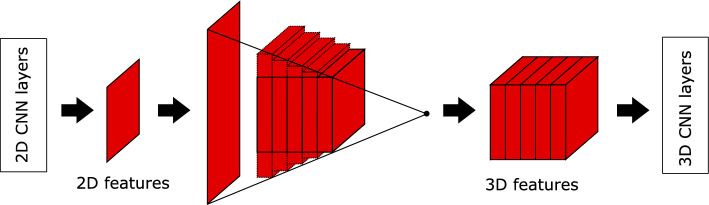
Illustration of the geometry-based fan-beam operator. In our network, such operator is implemented to simulate the propagation of 2D feature maps to 3D as backprojection.

One key step for the generator network is to convert the extracted features from 2D to 3D, and in our network such conversion is accomplished using backprojection, as the 2D X-ray projections are obtained by the forward projection of the CT volumes, as shown in Fig. 8. Fan-beam geometry is implemented in our research. The backprojection propagates the 2D feature maps to 3D and is implemented as a matrix multiplication,

5 $$\begin{aligned} \hat{Z}_{3D} = T \cdot \hat{Z}_{2D}, \end{aligned}$$

where $\hat{Z}$ is the flattened 2D or 3D intermediate feature maps and *T* is a pre-defined transformation matrix depending on the fan-beam geometry. In this work *T* is given by a pixel-driven fan-beam backprojector based on the geometry of a Siemens Somatom Force scanner.

### 3D segmentation loss

$L_s$ is first proposed for the enhancement of specific anatomical structures. The correct location, shape and size of the OARs in the reconstructed CT volumes are crucial for dose estimation and organ segmentation, but such anatomical content cannot be explicitly leveraged using typical image generation models, such as GANs. In order to include the organ segmentation into the training of the GAN, a dataset of CT volumes with the segmentation ground truth is required. However, the voxel-wise annotation of the OARs is very expensive in time and cannot be easily obtained for large-scale datasets for training a GAN, while the segmentation datasets are mostly not sufficient in the number of images for training GANs.

In our model, a pre-trained $\phi _s$ is leveraged to enhance the anatomical content that is missing in the reconstruction dataset. The $\phi _s$ is trained on an auxiliary dataset that contains the segmentation of the OARs in CT volumes. Such a $\phi _s$ is then applied into the training of the GAN and the enhancement of the anatomical structures is thus explicitly refactored to the optimization of OARs segmentation in the reconstructed CT based on the pre-trained $\phi _s$ . Such regularization is implemented as a loss item

6 $$\begin{aligned} L_s(y'_m, y_m)=1-2\frac{\sum y_my'_m}{\sum y_m + \sum y'_m} \end{aligned}$$

where $y_m=\phi _s(y)$ and $y'_m=\phi _s(G(x))$ are the organ segmentation mask of *y* and *G*(*x*) using $\phi _s$ . $L_s$ will depend on the target organ of $\phi _s$ , so the enhancement of anatomical structures can be targeted to specific organs. Since the segmentation ground truth of the reconstruction dataset is missing, $\phi _s$ will not be optimized during GAN training. After the training of the GAN, $\phi _s$ can be further used to provide organ segmentation. During the inference, the model outputs *G*(*x*) as reconstructed CT volume and $\phi _s(G(x))$ as the corresponding organ segmentation map with *x* being the input, i.e. the X-ray projections.

### 3D perceptual loss

Perceptual loss is first proposed in the field of computer vision for feed-forward image transformation tasks^16^. Unlike typical loss functions, perceptual loss relies on a pre-trained classification network as feature extractor and backpropagates the loss using the extracted features from the source and the target images. Apart from natural image researches, perceptual loss is also applied in medical image processing researches, such as the denoising of CT images^17^. It is shown that the network pre-trained on natural images can also work as a good feature extractor for medical images. In our model, we adopted the VGG-16 network pre-trained on the ImageNet dataset as the feature extractor, deployed by the torchvision toolkit (version 0.15.2)^18,19^. The original VGG-16 network contains five convolutional blocks to extract image features in different scales. The aggregation of L1 loss of intermediate features from the ground truth and the reconstructed CT volumes leads to the 3D perceptual loss, as shown in Fig. 6. The 3D perceptual loss used in the model training is

7 $$\begin{aligned} L_p(y', y)=\mathbb {E}_{y,y'}\left\| \phi _p(y)-\phi _p(y') \right\| _2^2, \end{aligned}$$

where the $\phi _p()$ is the intermediate features and only the first four VGG levels are used to aggregate the 3D perceptual loss. Note that the pre-trained VGG network only inputs 2D images, therefore the ground truth and the reconstructed CT volumes are sliced along the vertical direction and the loss of all 2D slices are aggregrated.

### Overall loss function

In addition to the previously mentioned loss functions, the voxel-based $L_{\text{r}}$ and pixel-based $L_{\text{proj}}$ are applied for the consistency of the input X-ray projections and the reconstructed CT volume, which is implemented as

8 $$\begin{aligned} L_{\text{r}}(y,y')&=\mathbb {E}_{y,y'}\left\| y-y'\right\| _2^2, \end{aligned}$$

9 $$\begin{aligned} L_{\text{proj}}(y,y')&=\frac{1}{2}\mathbb {E}_{y,y'}[\left\| P_{\mathrm {a.p.}}(y)-P_{\mathrm {a.p.}}(y')\right\| _2^2 + \left\| P_{\mathrm {lat.}}(y)-P_{\mathrm {lat.}}(y')\right\| _2^2], \end{aligned}$$

where $P_{\mathrm {a.p.}}$ and $P_{\text{lat}}$ project the CT volume each in a.p. and lat. direction. $L_r$ will lead to the CT reconstruction to be correct and $L_{proj}$ will lead to the projections to be correct. Then with the proposed $L_s$ and $L_p$ , the overall loss function for training the CT reconstruction GAN is weighted to balance the voxel-wise features and anatomical contents. The overall loss function aggregates as

10 $$\begin{aligned} L_D&= L_{\text{dis}} \end{aligned}$$

11 $$\begin{aligned} L_G&= \lambda _{\text{gen}}L_{\text{gen}} + \lambda _\text{r}L_{\text{r}} + \lambda _\text{proj}L_{\text{proj}} + \lambda _\text{s}L_{\text{s}} + \lambda _{\text{p}}L_{\text{p}} \end{aligned}$$

where $\lambda$s are configurable hyper-parameters. In the experiments we illustrated how the CT reconstruction is enhanced by the proposed model.

### Datasets

For the training of the GAN and the pre-training of the $\phi _s$ , two datasets are used in our experiment for the proof of the principle, i.e. a reconstruction dataset and a segmentation dataset. For the training of the GAN, the CT volumes from the lung image database consortium and image database resource initiative (LIDC-IDRI) are used as the reconstruction dataset^20^. The LIDC-IDRI dataset consists of 1016 chest CT volumes with pixel sizes ranging from 0.46 mm to 0.98 mm in the transverse plane and from 0.6 mm to 5.0 mm in vertical direction.

**Figure 9:**
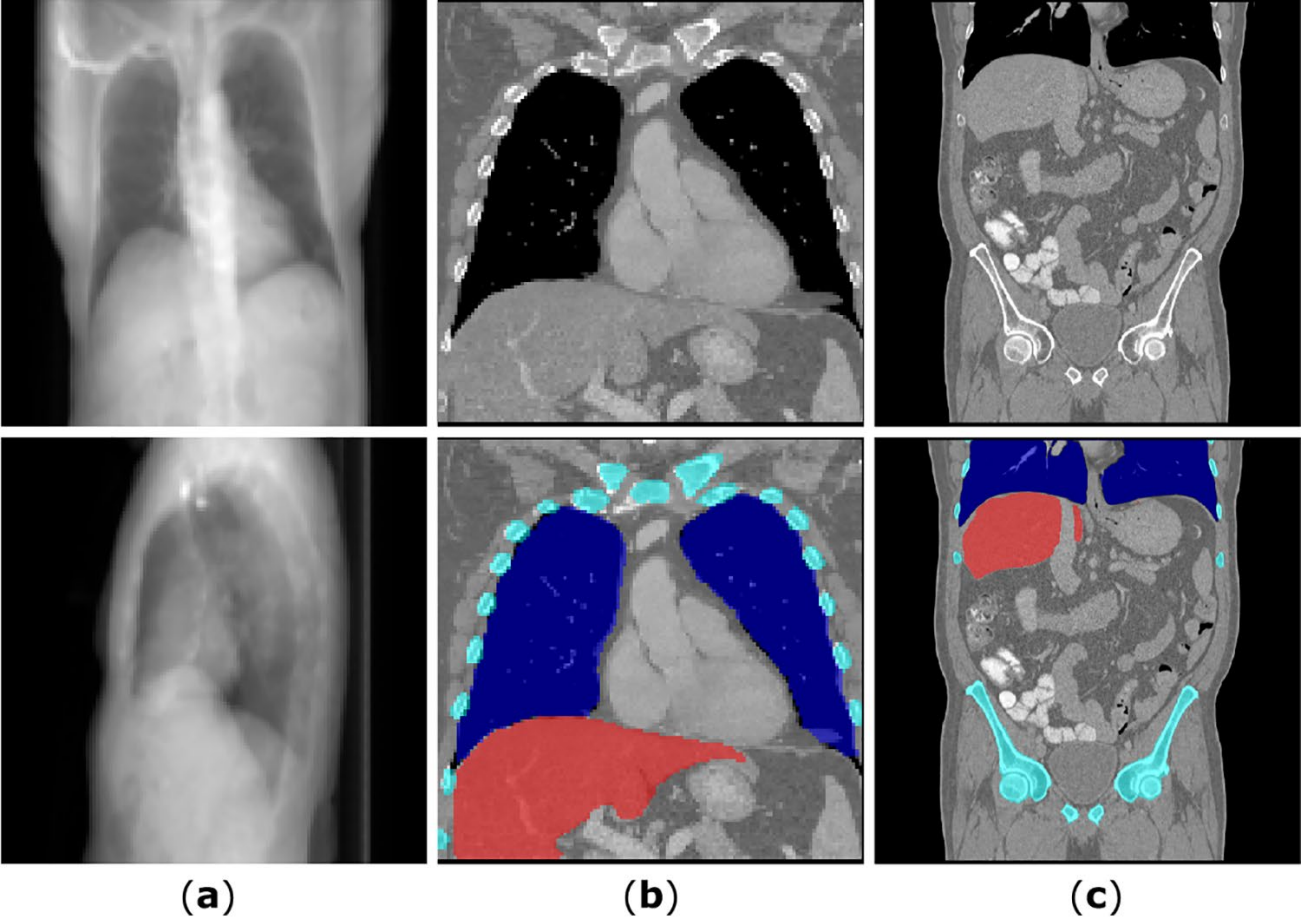
Example simulated X-ray projections and slices from CT volumes used in experiments. Column (**a**) shows the X-ray projections simulated by fan-beam geometry, column (**b**) shows the LIDC-IDRI dataset with manual organ annotation for evaluation and column (**c**) shows the CT-ORG dataset with organ annotation.

For the pre-training of the $\phi _s$ , we select a public dataset of CT volumes with voxel-wise annotation of abdominal organs, namely CT-ORG dataset^21^. The CT-ORG dataset consists of 140 throat-abdominal CT scans with annotated lungs, bones, liver, bladder and brain, with voxel sizes ranging from 0.56 mm to 1.0 mm in vertical direction. Because the reconstruction dataset covers only the chest region, the annotations of lungs, liver and bone in the CT-ORG dataset are used in the following experiments. Some samples from the datasets are shown in Fig. 9. All CT volumes in the LIDC-IDRI dataset and the CT-ORG dataset are resampled to a uniform voxel size of 1 mm by 1 mm by 1 mm to ensure the consistency during model training. 812 CT images from the LIDC-IDRI dataset are used during the model training and 214 images for testing. For training the $\phi _s$ , 112 CT scans are used for training and 28 images for testing. The X-ray projections are simulated in a way to mimic the fan-beam CT forward projection, by using the aforementioned scanner geometry. The CT volumes are first resampled to voxel size of 2.5 mm in each direction and then clipped to the uniform volume/image size of 128. The resolution of the input X-ray projections is also 128. For the GAN training, both X-ray projections and the CT volumes are normalized from 0.0 to 1.0 using the same parameters.

### Experiments

In all experiments, the GAN is trained for 100 epochs. Adam optimizer is used with the learning rate of $2\cdot 10^{-4}$. The weights of the GAN loss, the reconstruction loss and the projection loss, namely $\lambda _{\text{gen}}$, $\lambda _{\text{r}}$ and $\lambda _{\text{proj}}$, are fixed across all experiments, i.e. $\lambda _{\text{gen}}$ = 0.1, $\lambda _{\text{r}}$ = 10 and $\lambda _{\text{proj}}$ = 10. In all experiments, $\phi _s$ is implemented as an vanilla 3D U-Net as used in nnUNet^22^, containing five downsampling levels. $\phi _s$ is first trained on the CT-ORG dataset for 200 epochs. Dice loss is used as loss function and Adam is used as the optimizer with learning rate of $5\cdot 10^{-4}$. All model training is carried out on one Nvidia A100 GPU with 40GB memory. For the fan-beam operator, we modeled the real scanner parameters with the source-to-detector distance (SDD) as 1085.6 mm, the source-to-isocenter distance (SID) as 595 mm and the number of rays within the fan to be 920. Due to the relatively large size of training data and the high computational demand, cross-validation was not used.

## Data Availability

The LIDC-IDRI dataset used throughout the study also available via TCIA at https://wiki.cancerimagingarchive.net/x/rgAe. The CT-ORG dataset is available via TCIA at https://wiki.cancerimagingarchive.net/x/OgWkAw.
